# Use of welfare outcome information in three types of dairy farm inspection reports

**DOI:** 10.5713/ajas.17.0851

**Published:** 2018-04-12

**Authors:** Yi-Chun Lin, Siobhan Mullan, David C. J. Main

**Affiliations:** 1 School of Veterinary Sciences, University of Bristol, Langford House, Langford, Bristol BS40 5DU, UK; 2 School of Veterinary Medicine, National Taiwan University, Taipei 10617, Taiwan

**Keywords:** Animal Welfare, Cross Compliance Scheme, Farm Assurance Scheme, Outcome Measurement, Organic Standards, Dairy Cows

## Abstract

**Objective:**

The aim of this study was to examine the use of outcome-based observations within Assured Dairy Farm scheme (ADF), Soil Association Organic Standards (SA), and cross compliance (CC) farm assessment reports.

**Methods:**

A total of 449 ADF reports, 37 SA reports and 26 CC reports were analyzed and their objective comments categorized as either resource-based or outcome-based.

**Results:**

A mean of 61.0% of ADF questions were responded to with comments, in comparison to 25.0% of SA and, 21.0% of CC report questions. The SA and CC reports had significantly more outcome-based comments than the ADF (p<0.001). The assessors’ tendency of choosing resource-based approach was revealed in the questionnaire results.

**Conclusion:**

Generally, the comments were comprehensive and contained professional judgements. Large numbers of comments provided in the ADF reports were mostly compliant and resource-based evidence, which serves as proof of assessment rather than aiding the certifying process. The inclusion of specific welfare outcome measures in the SA inspection likely increased the use of outcome-based comments in the reports, irrespective of whether the farm achieved compliance with a given standards. The CC scheme, on the other hand, focused on providing outcome-based evidence to justify noncompliant decisions.

## INTRODUCTION

Since the outbreaks of diseases in the 1990’s, which affected food safety, the UK market established food assurance schemes aiming to ensure the safety of food to consumers. These private standards have extended beyond legal requirements and have increasingly responded to other public concerns, including animal welfare assurance [1].

Different approaches to assess animal welfare in a group level have been proposed [2]. Resource-based parameters have long been used to assess animal welfare [3]. These parameters include: i) facilities and environment that provided to the animals, ii) the feeding plans, iii) the vaccination programs that contribute to the physical health of the animals, iv) the stockmen competency, and v) animal factors that affect the welfare of the animal. These have been believed to be reliable, feasible and simple to record by the inspectors [4]. However, the provision of good resources does not guarantee good animal welfare [5]. It is seen as an indirect parameters in measuring animal welfare [6]. A major challenge for resource-based approach in certification scheme is that considerable variability in animal welfare may occur due to various farm enterprises [7].

Animal-based measures, on the other hand, are considered to be an approach that is directly measure the welfare of an animal [8]. Moreover, it was possible to benchmark farm performances among different farm systems [9]. The Farm Animal Welfare Council [10] recommended more animal-based outcome measurements should be included in assurance schemes for animal welfare. The outcome-based (animal-based) approach, as the effect of the resources, are direct measure of an animal, including directly observing the animals’ physical condition and behavior and may include examination of farm records [11]. The physical condition, such as body condition scoring, is a significant indicator of welfare status [12].

This study aimed to quantify the reporting of welfare outcomes following assessment of farms for three different reasons. Two schemes were included, the Assured Dairy Farms (ADF) Red Tractor scheme and the Soil Association Organic schemes (SA), both of which are commercial assurance schemes that use third party independent inspectors to assess against their standards. The other farm assessment reports analyzed were Government inspections for cross compliance (CC), which assess farms against the minimum EU legal requirements.

The Red Tractor Farm Assurance Dairy scheme, formerly known as the ADF covers the majority of UK dairy farms (95.0%). The core assurance for the ADF scheme is food hygiene and good quality to maintain consumer safety, with additional elements for animal welfare and environment protection. It sets its standard at or slightly above the legal minimum requirement. Major retailers in the UK require their supplies of dairy products be compliant with the ADF scheme. For those farms that are not compliant, either the farmers have 28 days to rectify the problem, or for more serious non-compliances, certification would be immediately suspended. In the latter case, farmers would be unlikely to be able to sell their products to the UK major retailers. Hence, in order to widen market access, farmers would have to be in accordance with the standards. In a sense, to be certified to the ADF standards is more or less compulsory for farmers.

The SA cover all aspects of food production and packaging, animal welfare, wildlife conservation, and regulates food additives in organic processed foods. The SA sets its standards based on the European Commission Organic Regulation, Council Regulation (EEC) No 2092/91, and above the legal requirement in some areas, for example, animal welfare and nature conservation.

The Soil Association Certification Ltd (SACL), an independent subsidiary of The Soil Association Charity, carries out inspections against the SA. To improve the quality of their inspections, SACL assessors were trained in the use of the Bristol Welfare Assurance Program (BWAP) protocol to provide further objective animal-based welfare evidence within their inspection reports [13]. The scheme operates similarly to the ADF when non-compliance exists. However, removal from the SA Organic certificates would not limit the access to all UK markets although the price of organic labelled food is higher. Organic farmers have more freedom in the choice of joining the SA scheme as they do so either due to the preference for organic farming systems or to capitalize on increased prices.

Cross compliance is a set of European Union (EU) requirements with which European farmers must comply in order to qualify for payment under the Common Agricultural Policy. These requirements cover public, animal and plant health, the environment and animal welfare. Competent Control Authorities, for example, in the UK, the Animal and Plant Health Agency, are responsible for determining the farms to assess and undertake the assessments of at least one per cent of farms.

Unlike the ADF and the SA Organic schemes, CC only checks whether farmers or landowners adhere to animal welfare legislation [14] and therefore CC has little influence in market access or price premium. However, for farmers who fail to comply, there is a penalty of a subsidy deduction from 15% to 100%. The amount depending on the level of severity of the breach.

The aim of this study was to use the written reports to determine the use of outcome measurements by the inspectors, and to understand their attitudes to outcome-based assessments through the use of a questionnaire.

## MATERIALS AND METHODS

In this study, three types of farm reports were analyzed for their use of outcome information, from the ADF scheme, checking government CC requirements and the SA organic scheme. Four certification bodies and one relevant authority were contacted and agreed to participate in the study. The reports were provided by each certification body via photocopied or electronic version. To minimize the bias, the criteria for selecting reports were even distribution from different assessors and 12-month if possible. The data collected were: i) the decisions of each question in the ADF, SA and CC farm reports regarding compliance, ii) the comments provided by the assessors, iii) the date of the report, iv) a code for each farm and assessors, to retain confidentiality. The date and the assessors’ details were not available in the CC reports. The categories that related to animal welfare, covered by each inspection are shown in Supplementary Table S1.

All the written comments that the assessors provided to support their judgement were classified into three groups, similar to the study of Keeling [4]. Firstly, any comment description that related to the environment provided to the cattle, the stockmanship, or the animal breed was classified as resource-based comments. Secondly, any comment described physical health and behavior from direct animal observations or farm records on disease prevalence were called outcome-based evidence. Thirdly, the comments, which were difficult to classify as the resource-based or the outcome-based, were classified as non-specific comments, e.g. ‘good welfare noted’. Some examples are shown in Supplementary Table S2.

### Assured dairy farm reports

Four-hundred and forty nine ADF reports from three third-certification bodies were analyzed. Due to a confidential agreement, these three certification bodies were coded as certification bodies A, B, and C. Certification body A provided 339 reports from annual assurance visits undertaken between April 2010 and March 2011. Certification Body B provided 60 reports that were undertaken between May 2010 and August 2010. Certification Body C provided 50 reports that were undertaken between April 2010 and April 2011.

The 166 questions in each ADF report were grouped into 17 different categories, as illustrated in Supplementary Table S1. The assessors could record whether compliant or not, and write a comment, for each question.

### Cross compliance

Twenty-six DEFRA Cross Compliance Statutory Management Requirement (SMR) 18 (Currently SMR 13) reports, namely the animal welfare requirement, for dairy farms were analyzed. Predominantly, the farms were selected from those expected to be at greater risk of non-compliance and a proportion of reports were randomly selected during 2010 to 2011. The reports consisted of 31 questions in 14 sections, are shown in Supplementary Table S1. The assessors could provide a score for compliance (A for compliance with legislation and codes, B for compliance with legislation but not code) and non-compliance (C for non-compliance but no adverse effect on the animal and D for evidence of unnecessary pain/distress) and defining the level of the breach severity, and sometimes, with comments for each category.

### Soil Association reports

Thirty-seven Soil Association Organic dairy inspection reports for dairy farms made between April 2010 and April 2011 were analyzed. The assessors were asked to provide an overall general comment and an average of 31 categories of compliance-non-compliance answers and supporting comments in reports (Supplementary Table S1). The categories range from 22 to 34, depending on different enterprises of farms. However, seven categories were excluded from this study because they did not relate to animal welfare, but organic compounds.

### Questionnaire

A 4-part questionnaire was designed to evaluate how assessors from the three certification bodies understood outcome comments (Supplementary Material S3). The questionnaires were distributed by the contacts of each certification body. The respondents were informed about the purpose of the survey and gave their consent to participate. The first part contained questions on the background of the assessors: the age, gender, the amount of years they had been working as auditors and their previous occupation. In part two assessors were asked to write down some examples statements that they would usually give to seven example questions that related to animal welfare taken from the ADF reports. Part three asked the assessors to define 11 commonly reported comments as either outcome-based or resource-based. The last part asked the assessors whether 2.7% (an estimated percentage of outcome comments out of total comments made from preliminary analysis of the first 100 ADF reports) of outcome usage in ADF reports was ‘too little’, ‘just right’ or ‘too much’ and asked them to provide reasons.

### Statistical analysis

A Pearson chi-square test performed using IBM SPSS Statistics Standard 19 (IBM SPSS Statistics for Windows, Version 19.0. Armonk, NY, USA: IBM Corp. Released in 2010) to establish the independency between the presence of a comment made and compliance/non-compliance decision and whether different comment types (outcome/resource-based) varied between compliance/non-compliance decisions, report categories, different time of the year, assessors, multiple group comparison was performed using the Kruskal-Wallis test for unequal sample size and pairwise post-hoc test was conducted when significant interactions were identified between certification bodies. Unclassified comments were not included in the analysis.

## RESULTS

From the 449 ADF reports, an average of 67.5±19.8 comments were made out of 111 questions in each report. A total of 30,240 (60.8%) questions were answered with a comment. Of these comments 29,189 (96.5%) were resource-based, 850 (2.8%) were outcome-based and 202 (0.7%) could not be classified as outcome or resource-based (Table 1).

The 26 CC reports contained a total of 806 questions with 286 comment boxes for the assessors to complete. An average of 2.3±2.2 comments were given out of 11 in each report. There were 60/286 (21.0%) comments made, of which 18/60 (30.0%) of them were outcome-based, 41/60 (68.3%) were resource-based and 1/60 (1.7%) comment could not be classified as either.

Within the 37 SA inspection reports, an average of 33±3.3 comments was contained in a report. A total of 1247 comments were made, of which 886 (71.1%) comments were resource-based comments, 320 (25.1%) were outcome-based comments and one (0.1%) was unable to be classified as outcome-based or resource-based.

Non-compliance rates in the three organizations varied. ADF had more non-compliant answers per farm. The three organizations usually provided a comment to support non-compliant decisions and CC (68.8%) provided a higher proportion of outcome-based comments compared to the other two organizations, 40.0% and 2.1% for SA and ADF respectively.

Supplementary Table S2 shows some example comments from reports relating to the three standards. Each example comprises the question from the standards, the comments and the decisions made by the assessors and the definition of the comment types.

There were significantly more comments made when ADF and CC standards were reported as non-compliant than compliant (ADF, p<0.001; CC, p<0.001). The structure of the SA reports meant it was not possible to apply this test to them considering the assessors provided comments for every question regardless of compliant or non-compliant decisions

A Kruskal-Wallis test showed that there was a statistically significant difference in using outcome approaches among the different standards (p<0.001). SA and CC reports had significantly more outcome-based comments than ADF reports (between ADF and SA p<0.001; between CC and ADF p< 0.001). The SA reports contained significantly more outcome-based comments for compliant decisions than non-compliant decisions (p<0.001). In contrast, we observed the CC reports contained more outcome-based comments for non-compliant decision than compliance, though not statistically significant (Table 1).

There were significant differences between comment type and different sections in ADF (p<0.001), CC (p = 0.006), and SA (p<0.001) farm reports.

**Figure 1:**
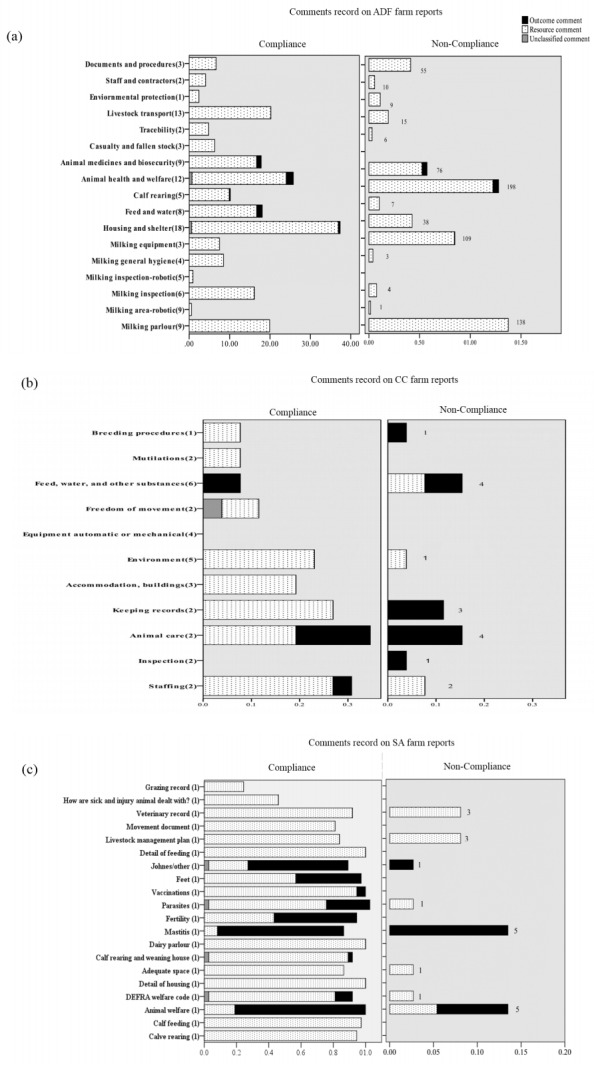
The mean number of comments made per farm report for sections where either all questions were compliant or one or more questions were non-compliant with the three standards. There are different numbers of question under different categories. The numbers of question under each section are marked in the brackets, except (C) as there is one question each under each section. Careful interpretation should be applied. The numbers marked on the right of each bar in non-compliance section are the total numbers of non-compliant decisions corresponding to each category.

Figure 1 shows a general summary of outcome/resource comments in various categories in the three standards. The numbers of questions in every category varied. To aid interpretation of results when there were different numbers of questions, the numbers in brackets behind each category indicate the number of questions in each section, and the numbers behind the bars in the ‘Not complied’ section show the total number of non-compliant questions made in each section.

In the ADF farm reports, ‘Animal Medicine and Bio-security’, ‘Feed and Water’ and ‘Animal Health and Welfare’ sections accounted for the most outcome-based comments, 281 (33.1% of outcome comments), 222 (26.1% of outcome comments) and 219 (25.8% of outcome comments), respectively. In the CC farm reports, eight (44.4% of outcome comments) cases of outcome comments were provided for the ‘Animal Care’ section, and four (22.2% of outcome comments) cases of outcome comments were for the ‘Feed and Water’ section. In the SA farm reports, the most outcome comments were found in five sections, namely ‘lameness’, ‘skin lesion’, ‘swollen hocks’, ‘coughing’ and ‘thin cows’, with 37 outcome comments (11.6% of all outcome comments) for each section. However, as these five parameters were not marked with compliance or non-compliance decisions, they were not included in Figure 1. The ‘Mastitis’ and ‘Animal welfare’ sections had 34 (10.6% of outcome comments) and 28 (8.8% of outcome comments) outcome comments, respectively.

ADF Farm reports from certification bodies A and C and SA organic standard farm reports related to assessments undertaken between April 2010 and April 2011. The time data with reports from certification body B was only available from May to August in 2010. There was no significant difference in the usage of outcome comments over the 12 different months in the reports of certification body A, C and SA (p = 0.781, p = 0.100, p = 0.972, respectively) or when data was grouped into 3-month periods from all three certification body (p = 0.372, p = 0.362, p = 0.659, respectively). No date data was available from CC farm reports.

The SA outcome measures were adapted from the BWAP protocols into the assessment procedure. The assessors were asked to observe 20 cows and record the number of cows on every farm that showed coughing behavior, lameness, skin lesions, swollen hocks and low body condition score. The mean prevalence for each measure from the 37 SA reports is shown in Table 2.

Within the ADF reports, there were 37 assessed farm reports that corresponded with SA farm reports. The total number of non-compliances within a report from both ADF and SA were summed up. In addition, the total number of animals affected by each of the five outcome measurements of the SA reports was summed up. The data was then reorganized into binary categories for presence or absence of a non-compliance, and similarly for any animals affected by a welfare outcome score on a farm. A Pearson’s Chi-square test was carried out to examine the independency of these variables. The results showed that the presence of non-compliance in ADF and SA reports were independent from the presence of any five-outcome-measurements (p = 1.000 for ADF and p = 0.963 for SA). Table 3 shows that the number of both ADF and SA reports with any non-compliance decision which contained at least one five-outcome-measurements is similar to the reports of which containing no affected condition.

### Questionnaire

There were a total of 35 surveys completed by assessors from the ADF scheme, 11 from certification body A, 10 from certification body B and 14 from the certification body C. Twenty-five assessors completed the paper version of the questionnaire and ten assessors completed the questionnaire online.

Male assessors were the majority (94.3%), they were a range of ages but most commonly over 60 years old (10 assessors) and most had more than three years’ experience of assessing farms (94.3%).

Assessors most commonly reported they would use outcome comments for the question of ‘Are the welfare needs of the stock met at all times?’ (Table 4). Twenty-one assessors had written down outcome comments such as, ‘Look at positive and negative aspect or cattle behavior as well as the physical aspect’, ‘Record if cattle are quiet/calm and if any sign of injury or distress seen at time of visit’, or 'Describe system of …, refer to BWAP criteria, access to feed/water, housing/cleanliness of stock, body condition, mortality’.

The fewest outcome comments were for the question of ‘Are all stock provided with sufficient access to feed?’ Three assessors suggested they would measure body condition, but most of the assessors reported they would measure the length of feed areas and number of places provided for the herds. The former comments would be defined as outcome-based comments, and the latter comments would be resource-based comments in this study.

Assessors were also asked to define whether examples of objective evidence given were outcome-based or resource-based, as shown in Supplementary Table S4.

**Figure 2:**
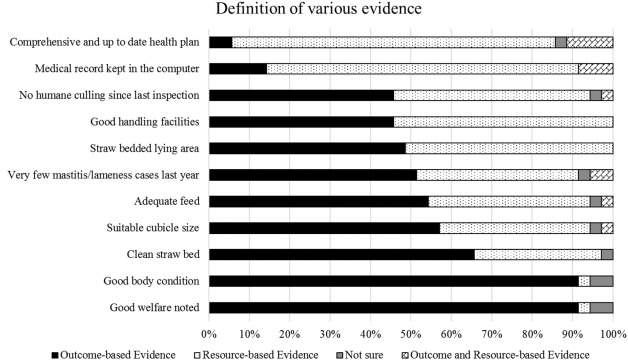
Assessors’ definition of various comments. The descriptions were chosen as the ones which often appeared on the farm reports. The assessors were asked to express their interpretations of the descriptions into ‘outcome-based evidence’ category or ‘resource-based evidence’ category. The majority of the assessors agreed that ‘good welfare noted’ and ‘good body condition’ as outcome-based evidence. Most of the assessors considered the description of ‘medical record kept in the computer’ and ‘comprehensive and up to date health plan’ were resource-based evidence. The interpretations of the other descriptions were inconsistent among the assessors.

The majority, 32 people out of 35, thought that ‘Good welfare noted’ and ‘Good body condition’ were outcome-based evidence, only one assessor thought they were resource-based and two were not sure. The evidence that fewest assessors (two people) rated as outcome-based was ‘Comprehensive and up to date health plan’ and most of them, 28 out of 35, defined it as a resource-based comment (Figure 2).

Most assessors, 23 out of 35, thought that 2.7% of outcome comments made per reports from ADF assessments was too little. Six of them thought it was the right level in reports, but two assessors claimed that it was too much for the ADF assessment. Moreover, some assessors argued that some of the questions, which are in the reports, are repeated in different sections and some suggested that some questions need to be rephrased to be clear what exactly the assessment should be focusing on. However, the data were insufficient to test the independency of assessors’ orientation, whether they were resource or outcome orientated, against the definitions that they gave in part three.

## DISCUSSION

The private assurance scheme standards operated by licensing the certification bodies to conduct certifying process. Third party inspection is often being applied to ensure there is no conflict of interest [15]. The certification bodies must undergo accreditation by the United Kingdom Accreditation Service to EN ISO/IEC 17065:2012. Based on this requirement, the certification bodies are responsible for providing sufficient objective evidence to support the decision. The comments provided by the assessors, in this study, are considered by the certification bodies as the objective evidence. Whether the evidence directly reveal the animal welfare status to support the certification process or whether they promote better animal welfare became the main focus of this study. By comparing the three types of reports, it was found that there were distinct features of each scheme. The ADF farm reports contained vast amounts of comments, approximately three times those in the SA reports, and nearly thirty times the CC farm report comments. However, the resource-based comments still occupied a major proportion in the certification process in the ADF. The ADF assessors were asked to check/fill more questions than the CC inspectors and the SA assessors within a similar time scale. For example, while the three standards all covered whether feed is enough to maintain health and whether there is adequate access to feed and water, the questions used to ascertain this information were structured differently.

Sections related to feed and water in the SA farm reports were mostly checking the resource of feed being organic compounds and to assess the animal welfare aspect, the SA assessors used body condition scoring of 20 cows for sufficiency of diet, under the ‘thin cow’ section. In the CC farm reports, three questions were asked about the sufficiency and accessibility of diet/water and two questions were about whether feed/water or feeding/watering equipment was causing stock any unnecessary pain and suffering, all of which are based on Council Directive 98/58/EC of 20 July 1998 concerning the protection of animals kept for farming purposes. The ADF farm reports contained five questions covering sufficiency and accessibility of feed and water, in a similar way to the CC farm reports. However, there were ten more questions based on The Animal Feed (England) Regulations 2010 for feed hygiene reflecting the stated major aim of ADF assurance being food safety.

Another interpretation for the large proportion of resource-based comments might be that some resource-based measures are seen to be more practical and less subjective [16,17]. A focus group study found that assessors thought the problem with outcome-based measures was that they were time-consuming [18].

Although the SA Organic assessors had a similar background to the ADF assessors the outcome comment percentage (25.0%) in the SA inspection reports was still higher than in the ADF reports particularly in compliant rather than in non-compliant decisions. Clear guidelines promoting the use of outcome comments in the reports might account for the higher proportion of outcome comments. Furthermore, training to develop the techniques of outcome assessments following the BWAP were provided to SA assessors [13] to enable the assessors to focus more on observing the animals that might also explain the high percentage of outcome comments in the SA inspection reports.

Despite having fewer comments made in the CC reports, it was significant that a higher proportion of outcome comment were contained in the CC reports. In most cases, the CC inspectors did not comment unless there was a breach of the standard when inspectors would provide comprehensive comments containing professional judgement, describing in detail the exact nature of the non-compliance using a combination of resource-based and outcome-based observations.

Due to the difference in private schemes and legislation, the work instruction for assessors/inspectors, may play a big role in their styles of writing reports. Certification bodies must provide evidence to demonstrate the effectiveness of the certification procedure. However, the CC assessors have more freedom than private farm assurance assessors in commenting in the reports, and guidance encourages them to focus on non-compliant decisions. They also have fewer questions to complete in a similar time frame. It might reflect differences in the use of animal-based evidence. Further research into the guidance and the training materials might be beneficial in identifying the assessors’ weakness and developing an assessor training strategy.

The prevalence of five outcome measures from the SA reports were lower than other existing studies [19–21]. One possible interpretation might be that organic farms have higher animal welfare standards than non-organic ones [22,23]. However, prevalences reported for outcome measures were often higher than those found in the SA reports [24–26]. For instance, based on the results of Rutherford et al [26], the average prevalence of poor body condition (less than body condition score 2) was 18.9% in organic and non-organic farms (range from one per cent to 47.4%) and the mean lameness prevalence was estimated around 24% in the organic farms in the UK [27]. A similar study from Barker et al [19] showed that the mean lameness prevalence was 36.8% with nearly one third of organic farms being included in the data collection. A second possible explanation might be that assessors are less sensitive to the outcome measures as Mullan et al [28] found that observer bias might lead to assessors being less sensitive when identifying problems. A third possible interpretation might be that assessors might not be willing to record welfare problems to avoid confrontation. It was highlighted in the study of Anneberg et al [29] that some farmers do not appreciate it when the assessors ‘give them a lesson’ during inspections, and one of the assessors described the relationship between themselves and farmers as ‘a knife-edge balance’.

In the questionnaire section, the assessors were asked to complete seven questions from the Red Tractor Assurance (RTA) standards based on their experience. Those questions, in the authors’ opinion, have the potential to include animal-based comments. However, the results shown in Table 4 indicated that most assessors would use resource-based comments in assessing areas regarding these seven questions. The reasons might be that either they were more comfortable using resource-based comments or they were not sure it was possible to include animal-based comments in those questions. These in turn may have depended on previous training (veterinary for CC and formal animal-based observations for SA). Since this study was conducted, welfare outcome assessments have been expanded on The SA dairy inspections accompanied by additional training (AssureWel.org.uk). The ADF scheme has implemented welfare outcome assessments on 10 cows on each herd for lameness, lesions, swellings, dirtiness and thin/fat cows, as well as collecting records-based information about mortality, lameness and mastitis. These assessments are designed to support compliance decisions and further work could be undertaken to assess whether there has been an increase in outcome usage since the introduction of formal welfare outcome assessments.

Upon requesting the reports, some certification bodies archived their reports electronically, yet, still some kept the written reports in a paper format. The latter increased the effort in obtaining equal sample size, which therefore presents a study limitation. These limitations were accounted for in analyses.

Another limitation of this study relates to the dissimilar time frame of the reports. One cluster of the reports were completed within a four-month period. The time frame was not provided with CC reports, either. Nevertheless, the time factor was not influential for the assessor to choose between resource-based and outcome-based approaches, as no significance could be observed with the farm reports from certification body A, C, and SA.

Furthermore, assessors’ auditing styles could affect the approaches in completing the farm reports. Hence, evenly distributed assessors were specified, upon requesting the reports from the certification bodies to avoid potential bias.

Each of the limitations above might lead to some bias in our analysis. The main intention of this study, however, was to acquire a better understanding on the tendency of different approaches that being used in the animal welfare assessments. The results highlighted the habit of using resource-based methods in commercial farm assessments and the importance of training required for the assessors to perform outcome-based approaches with confidence.

## CONCLUSION

Many policy makers have encouraged the use of outcome-based measurements in farm assurance schemes as a more direct indicator for identifying animal welfare status [30]. Moreover, an effective practice of welfare certification schemes proposed by Main et al [2] is a combination of resource and outcome approaches. This study showed that outcome-based comments were used more in the CC scheme, which is direct legislation assessment, followed by the SA. Resource-based comments were much higher in the ADF assurance scheme. The CC inspectors were more likely to make their decisions based on outcome measurements, although the CC inspectors made fewer comments compared to the SA and the ADF assessors. Moreover, the evidence provided by the CC inspectors in non-compliant decisions in particular was comprehensive and detailed. The question of whether it is necessary to write general comments for the vast amount of questions within the standards or just complete in detail when there are breaches. It might be preferable within a limited time scale to allow all assessors to focus on more valuable non-compliance comments. An in-depth interview approach might be beneficial in exploring specific and individual rationale in opting for different measures in corresponding to each criterion in the future.

## IMPLICATIONS

As the awareness of animal welfare has increased with the public, the food certifying schemes could play a role to improve animal welfare gradually. Moreover, a frequent review of the assurance scheme standards to keep up to date with the scientific research may be the direction for animal welfare improvement. This study was the first to compare the usage of outcome measurements in animal welfare standards within the UK private food certification schemes and the European Union (EU) legislation requirements. This study may provide advice in developing animal welfare certification schemes for market segmentation in the future.

## Supplementary Data



## Figures and Tables

**Table 1:** The compliance rates and use of comments in the ADF, SA, and CC reports

Standards	Total number of questions	NM (N)	PC (%)	NOC (N)	POC (%)	NRC (N)	PRC (%)	NMR (N)	NOCR (N)	NRCR (N)
Compliance										
ADF	42,026	28,452	67.7	829	2.9	27,422	96.4	63.37	1.85	61.07
CC	655	44	16.9	7	2.7	36	13.8	1.69	0.27	1.38
SA	989	989	100.0	125	12.6	864	87.4	26.73	3.38	23.35
Non-compliance										
ADF	750	734	97.8	17	2.3	717	97.7	1.63	0.04	1.60
CC	16	16	100.0	11	68.8	5	31.3	0.62	0.42	0.19
SA	20	20	100.0	8	40.0	12	60.0	0.54	0.22	0.32

ADF, Assured Dairy Farm scheme; SA, Soil Association Organic Standards; CC, cross compliance; NM, number of comments made; PC, percentage of comments; NOC, number of outcome comments; POC, percentage of outcome comments; NRC, number of resource comments; PRC, percentage of resource comments; NMR, number of comments made per report; NOCR, number of outcome-based comments made per report; NRCR, number of resource-based comments made per report.

**Table 2:** A table of spot proportion of outcome measurements from 37 Soil Association Organic farm assessment reports

Outcome measurement	Prevalence (%)
Coughing	0 (range from 0.0% to 0.0%)
Lameness	1.8 (range from 0.0% to 15.0%)
Skin lesion	2.4 (range from 0.0% to 25.0%)
Swollen hocks	0.7 (range from 0.0% to 20.0%)
Low body condition score	0.7 (range from 0.0% to 5.0%)

**Table 3:** A table of the number of reports with the presence of any five-outcome-measurements against the presence of non-compliance in both SA and ADF reports

Item		The presence of non-compliance in SA reports			The presence of non-compliance in ADF reports		
	
		Com	NCom	Total	Com	NCom	Total
The presence of any five-outcome-measurements	Not affected	13	7	20	9	11	20
	Affected	10	7	17	8	9	17
	Total	23	14	37	17	20	37

SA, Soil Association Organic Standards; ADF, Assured Dairy Farm scheme; Com, compliance; NCom, non-compliance.

**Table 4:** A summary table of the comments classification that were reported by the assessors for seven potential outcome assessment questions

Questions from Assured Dairy Farm scheme standards	Outcome comments	Resource comments	Unclassified comments
‘Are the welfare needs of the stock met at all times?’	21	6	7
‘Is there an up to date medicine record containing all of the requirements of the current standard?’	19	14	2
‘Is a review of the health plan, including a collation of the number of cases of lameness and mastitis and culling rate, carried out at least annually?’	18	15	2
‘Are medicines only used when necessary and according to current legislation?’	8	24	2
‘Are cubicle systems appropriately designed to allow cattle to exhibit normal behavior with at least 1 cubicle per cow and adequate loafing area?’	7	24	2
‘Is housing of sufficient size to allow for appropriate group size and stocking densities?’	5	29	1
‘Are all stock provided with sufficient access to feed?’	3	30	2
Total	81	142	18
