# Supplementary material for: Use of welfare outcome information in three types of dairy farm inspection reports
Source: Asian-Australas J Anim Sci. 2018 Apr 12;31(9):1525–34. doi: 10.5713/ajas.17.0851 (PMC6127581; doi:10.5713/ajas.17.0851)
Supplement: Supplementary file 1 [file ajas-31-9-1525-supplementary.pdf]

Use of welfare outcome information in three types of dairy farm inspection reports

Y. Lin, S. Mullan, and D. C. J. Main

SUPPLEMENTARY MATERIAL

Supplementary Table S1. The different categories from the three standards that relate to animal welfare which were included in the analysis.

Categories to assess within the standards	Assured Dairy Farm	Cross Compliance	Soil Association Organic Standards
1	Staff and contractors	Staffing	Animal welfare
2	Animal health and welfare	Animal care	How are sick and injured animals dealt with?
3	Animal medicines and biosecurity	Keeping records	Veterinary records
4	Calf rearing	Accommodation buildings	Mastitis
5	Environmental protection	Environment	Parasites
6	Housing and shelter	Freedom of movement	Johnés/other
7	Feed and water	Feed, water and other substances	Vaccinations
8	Livestock transport	Breeding procedures	Adequate space
9	Milking - general hygiene	Equipment automatic or mechanical	Details of housing
10	Milking area-robotic	Mutilations	Calves rearing
11	Milking equipment	SMRS 13, 14, 15 (control of foot and mouth disease, certain animal diseases and bluetongue)	Calf feeding
12	Milking inspection	Inspection	Livestock management plans
13	Milking inspection-robotic		Dirtiness - welfare spot only
14	Milking parlour		Lameness
15	Traceability		Skin lesions
16	Casualty and fallen stock		Swollen hocks
17	Documents and procedures		Thin cows
18			Coughing
19			Fertility

20	Defra welfare codes
21	Grazing records
22	Dairy parlour
23	Details of feeding
24	Calf rearing and weaning housing
25	Movement documentation
26	Mortality/rejection records
27	Stock/bird movements
28	Feet

7

Supplementary Table S2. Some examples of the comments made by assurance assessors from the farm reports of the three schemes and their classification by the author.

Ex.		Question	Comment	Compliance status	Comment Classification
1	ADF	Is housing constructed to provide a safe, hygienic and comfortable environment for stock and maintained to avoid injury and distress?	Good housing. /All appear satisfactory to good.	Compliant	Resource
2	ADF	Is housing of sufficient size for the appropriate stocking density to be met?	Adequate stock numbers. Spare stalls.	Compliance	Resource
3	ADF	Do stocks receive a daily diet sufficient to maintain full health and vigour?	Cattle look well. / All stock in good condition & healthy. /All cattle appear in good condition, condition score 3 to 3.5 in cows. /Stock in good body condition, all look bright and healthy.	Compliance	Outcome
4	ADF	Is a review of the health plan, including a collation of the number of cases of lameness and mastitis and culling rate, carried out at least annually?	Annually-Mastitis-5 cases, Lameness-2 cases. 2 per year. /28 cases mastitis, 0 cases Lameness 20 % cull rate.	Compliance	Outcome
5	ADF	Are detailed medicine records for all treatments and medicines purchased and administered, including those administered by the vet, kept updated and retained for at least five years?	Insufficient information being kept for medicine usage - no meat or milk withdrawal end dates, no batch number recorded against medicine used, no mention of who administered.	Non-compliant	Outcome

6	ADF	Is a written health plan established, implemented and reviewed regularly according to circumstances?	BCVA plan last reviewed on 01/09/09. Herd health plan has not been reviewed with the past 12 months.	Non-compliance	Resource
7	ADF	Are cubicle systems designed to allow cattle to exhibit normal behaviour?	Super comfort cubicles. 1	Compliance	Unclear
8	ADF	Are the welfare needs of the stock met at all times?	No welfare issues noted. 2	Compliance	Unclear
9	CC	Are sick animals suitably cared for and where necessary getting veterinary advice? /Is accommodation for sick and injured animals suitable for purpose?	Regular visits from vets. 3 chronic lame cows in yard awaiting Owner. One lame cow to be shot left in cubicle house as unable to rise this morning but in sternal recumbence & comfortable. When seen had collapsed in passageway so I euthanized it. I confirmed that owner had been arranged as claimed & also confirmed with vet practice that this cow was up, ambulatory and giving no serious cause for concern at bTB test 2 days before so no breach.	Compliance	Outcome
10	CC	Are sick animals suitably cared for and where necessary getting veterinary advice? /Is accommodation for sick and injured animals suitable for purpose?	There was a high incidence of severely lame cows that had not been effectively treated. He has not consulted his vet or foot trimmer and has not been foot bathing as previously advised. This is an intentional breach as he has been given a lot of advice about lame cows over several visits and a prosecution for	Non-compliance	Outcome

			causing suffering by failing to treat lame cows was pending at the time of this visit.		
11	SA	Details of housing	Cubicles and loose housing. Cubicles with rubber mats bedded with untreated sawdust. Straw bedding for loose housing. Slatted passageways in new building.	Compliance	Resource
12	SA	Livestock Management Plans	BCVA Health Planner initially compiled 27/06/05 and last reviewed 15/09/10. As detailed above, this plan does not detail specific treatments administered to cows with toxic mastitis or acute lameness. These elements of the plan are to be reviewed again.	Non-compliance	Outcome
13	SA	Lameness/ Swollen hocks/ coughing/ skin lesion/ thin cow	3 out of 20.	N/A	Outcome

[1] With the comment of ‘super comfort cubicle’, the word ‘comfort’ confused the author. The author had difficulties to learn that whether it is describing the adequate space, straw quality or mat depth in the cubicle or the cow feels comfortable inside the cubicle.

[2] In regard to the comment of ‘no welfare issue noted’, the author also had difficulties classifying it to the correct category. Animal welfare issue can be identified by either or both outcome-based and resource-based measurements. Without clearly stated in which areas the assessors were judging from, the comment could only serve as a yes or no answer to that specific question.

Supplementary Material S3. Questionnaire on objective evidence in Farm Assurance Report

This survey is part of the on-going project to study about Farm Assurance Scheme in dairy cattle. The information that you provide is essential to find out the efficiency of the reports. This questionnaire takes about 10-15 minutes to complete. Thank you for your participation.

1. Which age group are you? □20-30 □ 30-40 □40-50 □50-60 □Above 60

2. What is your gender? □Male □Female

3. How long have you been an assessor?

   □Less than 3 years□3-6 years□6-9 years □More than 9 years

4. What was your previous occupation? ....

5. Which scheme(s) do you assess? ……………………………………….

6. What objective evidence do you normally record in the comments box when assessing the following standard?

A	‘Is housing of sufficient size to allow for appropriate group size and stocking densities?’	
B	‘Are cubicle systems appropriately designed to allow cattle to exhibit normal behaviour with at least 1 cubicle per cow and adequate loafing area?’	
C	‘Are all stock provided with sufficient access to feed?’	
D	‘Are the welfare needs of the stock met at all times?’	
E	‘Is a review of the health plan, including a collation of the number of cases of lameness and mastitis and culling rate, carried out at least annually?’	
F	‘Are medicines only used when necessary and according to current legislation?’	
G	‘Is there an up to date medicine record containing all of the requirements of the current standard?’	

7. Do you think the following evidence is outcome or resource based? (Please tick the box)

	Outcome-based	Resource -	Not sure

	evidence based evidence
Medical record kept in the computer	
Very few mastitis/lameness cases last year	
Clean straw bed	
Adequate feed	
Good body condition	
Suitable cubicle size	
Comprehensive and up to date health plan	
No humane culling since last inspection	
Good handling facilities	
Good welfare noted	
Straw bedded lying area	

8. We looked at 111 animal health or welfare related questions on 30 ADF reports, 2386 out of 3330 (71.7%) questions included objective evidence comments, 2306 (96.6%) of these were resource-based comments, 64 (2.7%) were outcome-based and 16 (0.7%) were the combination of two.

A	Do you think this level of outcome-based evidence is: (Please tick the box)	Too little ☐	Just right ☐	Too much ☐	Not sure ☐
B	Please explain why you think the result is too little/ just right/too much. Are there any difficulties for assessing outcomes?				
C	If you think there should be a change in the use of outcome-based objective evidence within the ADF reports please comment on how this could be achieved.				

Supplementary Table S4. A summary table of comment definitions from the authors and most of the assessors.

Example comments	Author's definition	Most assessors' definition
Good welfare noted	Unclassified	Animal-based
Good body condition	Animal-based	Animal-based
Clean straw bed	Resource-based	Animal-based
Suitable cubicle size	Resource-based	Animal-based
Adequate feed	Resource-based	Animal-based
Very few mastitis/lameness cases last year	Animal-based	Animal-based
Straw bedded lying area	Resource-based	Resource-based
Good Handling facilities	Resource-based	Resource-based
No humane culling since last inspection	Animal-based	Resource-based
Medical record kept in the computer	Resource-based	Resource-based
Comprehensive and up to date health plan	Resource-based	Resource-based
